# Subinhibitory Concentrations of Triclosan Promote *Streptococcus mutans* Biofilm Formation and Adherence to Oral Epithelial Cells

**DOI:** 10.1371/journal.pone.0089059

**Published:** 2014-02-13

**Authors:** Telma Blanca Lombardo Bedran, Louis Grignon, Denise Palomari Spolidorio, Daniel Grenier

**Affiliations:** 1 Department of Oral Diagnosis and Surgery, Araraquara Dental School, State University of São Paulo, São Paulo, Brazil; 2 Oral Ecology Research Group, Faculty of Dentistry, Université Laval, Quebec City, Quebec, Canada; 3 Department of Physiology and Pathology, Araraquara Dental School, State University of São Paulo, São Paulo, Brazil; University of Oklahoma Health Sciences Center, United States of America

## Abstract

Triclosan is a general membrane-active agent with a broad-spectrum antimicrobial activity that is commonly used in oral care products. In this study, we investigated the effect of sub-minimum inhibitory concentrations (MICs) of triclosan on the capacity of the cariogenic bacterium *Streptococcus mutans* to form biofilm and adhere to oral epithelial cells. As quantified by crystal violet staining, biofilm formation by two reference strains of *S. mutans* was dose-dependently promoted, in the range of 2.2- to 6.2-fold, by 1/2 and 1/4 MIC of triclosan. Observations by scanning electron microscopy revealed the presence of a dense biofilm attached to the polystyrene surface. Growth of *S. mutans* in the presence of triclosan at sub-MICs also increased its capacity to adhere to a monolayer of gingival epithelial cells. The expression of several genes involved in adherence and biofilm formation in *S. mutans* was investigated by quantitative RT-PCR. It was found that sub-MICs of triclosan significantly increased the expression of *comD*, *gtfC*, and *luxS*, and to a lesser extent of *gtfB* and *atlA* genes. These findings stress the importance of maintaining effective bactericidal concentrations of therapeutic triclosan since sub-MICs may promote colonization of the oral cavity by *S. mutans*.

## Introduction

Triclosan (2,4,4′-trichloro-2′-hydroxydiphenyl ether) is a non-ionic molecule with broad-spectrum antimicrobial activities against both bacteria (Gram positive and negative) and fungi [1]. More specifically, it is a general membrane-active agent that causes structural perturbations resulting in a loss of permeability-barrier functions [2]. Triclosan has also been reported to possess an anti-inflammatory activity since it reduces cytokine secretion by host cells such as gingival fibroblasts [3], [4]. Given this interesting dual action associated to triclosan and the fact that its high substantivity can be associated to a long-lasting effect, it has been used in oral care products (mouthwash, toothpaste) as an active agent for the reduction of dental plaque accumulation and the control of inflammatory gingivitis. The systematic review performed by Davies *et al.* [5] supports the benefits of triclosan-containing toothpaste for reduction of dental plaque and gingivitis. Being a general anti-plaque agent, triclosan may also be effective for the management of dental caries, a chronic infectious disease associated with a progressive destruction of the hard tooth structures (enamel, dentine, cementum) by the action of acidogenic/aciduric bacteria, mainly *Streptococcus mutans*, embedded in the dental biofilm [6], [7]. To support that, it has been reported that triclosan-containing toothpastes enhance the anti-caries potential of fluoride in dentifrices [8]. Phan and Marquis [9] brought evidence that the ability of triclosan to inhibit glycolysis of *S. mutans* in biofilms may be, at least in part, responsible for its anti-caries effect.

The effective therapeutic results of antimicrobials are optimal when the concentration is above the minimum inhibitory concentration (MIC). Several studies have reported that antimicrobials at sub-MICs may modulate biological characteristics of bacteria, including their ability to colonize the host [10]–[15]. In this study, we investigated the effect of triclosan sub-MICs on *S. mutans* biofilm formation and adherence to oral epithelial cells. In addition, the effect of triclosan at sub-MICs on expression of several genes involved in adherence and biofilm formation was investigated.

## Materials and Methods

### Bacteria and Growth Conditions

*S. mutans* ATCC 25175 (serotype c) and ATCC 35668 (unknown) were used in this study. Bacteria were grown aerobically at 37°C in Todd-Hewitt broth (BBL Microbiology Systems, Cockeysville, MD, USA) supplemented with hemin (10 µg/ml) and vitamin K (10 µg/ml) (THB-HK).

### Determination of MIC of Triclosan

Overnight cultures of *S. mutans* were diluted in THB-HK to obtain an optical density at 660 nm (OD_660_) of 0.2 (corresponding to 1×10^8^ colony-forming units [CFU]/ml). Samples (100 µl) were added to the wells of a 96-well tissue culture plate containing serial dilutions (125 to 0.50 µg/ml) of triclosan (Sigma-Aldrich Canada Ltd., Oakville, ON, Canada) in culture medium (100 µl). Control bacteria were cultivated in the absence of triclosan. After incubation for 24 h at 37°C, bacterial growth was monitored by recording the OD_660_ using a microplate reader. The MIC was defined as the lowest concentration of triclosan that completely inhibits *S. mutans* growth (OD_660_≤0.05).

### Biofilm Assay

*S. mutans* was grown (24 h) in a flat-bottomed 96-well microplate as above in the absence (control) and presence of triclosan at 1/2, 1/4, or 1/8 MIC. Similar assays were also performed using THB-HK supplemented with 0.25% sucrose. The medium, free-floating bacteria, and loosely-bound biofilm were then removed by aspiration, and the wells were washed three times with 50 mM phosphate-buffered saline (pH 7.2; PBS). The biofilms were stained with 0.04% crystal violet (100 µl) for 10 min. The wells were washed three times with PBS to remove unbound crystal violet dye and dried for 2 h at 37°C. After adding 100 µl of 95% (v/v) ethanol to each well, the plate was shaken for 10 min to release the dye from the biofilms and the absorbance at 550 nm (A_550_) was recorded.

### Scanning Electron Microscopy

The *S. mutans* (ATCC 25175) biofilm was examined by scanning electron microscopy. One ml of *S. mutans* resuspended to an OD_660_ of 0.1 in culture THB-HK ± triclosan at 1/2 or 1/4 MIC was added into wells of a 6-well plate containing a 13 mm-diameter plastic coverslip. After 24 h incubation, medium and free-floating bacteria were removed. The biofilms were incubated overnight in fixation buffer (4% (w/v) paraformaldehyde, 2.5% (w/v) glutaraldehyde, 2 mM CaCl_2_ in 0.2 M cacodylate buffer, pH 7.2), washed with 0.1 M cacodylate buffer pH 7.0 (3×20 min) and post-fixed for 90 min at room temperature in 1% (w/v) osmic acid containing 2 mM potassium ferrocyanide and 6% (w/v) sucrose in cacodylate buffer. Samples were dehydrated through a graded series of ethanol (50, 70, 95 and 100%), critical point dried, gold sputtered and examined using a JEOL JSM6360LV scanning electron microscope operating at 30 kV.

### Assay for Adherence to Oral Epithelial Cells

*S. mutans* ATCC 25175 cells cultivated in the absence (control) or presence of triclosan at 1/2, 1/4 or 1/8 MIC were labeled with fluorescein isothyocyanate (FITC) as previously reported [16]. The immortalized human gingival epithelial cell line OBA-9 used in this study, kindly provided by Dr. Marcia Mayer (Departamento de Microbiologia, Institute of Biomedical Sciences, Universidade de São Paulo, São Paulo, Brazil), was initially described by Kusumoto *et al.* [17]. The epithelial cells were cultured (96-well microplate) in Keratinocyte-Serum Free Medium (K-SFM, Life Technologies Inc., Burlington, ON, Canada) containing insulin, epidermal growth factor, and fibroblast growth factor, and supplemented with 100 µg/ml of penicillin G/streptomycin at 37°C in a 5% CO_2_ atmosphere until they reached confluence. The adherence assay of *S. mutans* to epithelial cells was carried out as described in a previous study [16]. After removing unbound bacteria and washing wells, the relative fluorescence units (RUF; excitation wavelength 495 nm; emission wavelength 525 nm) corresponding to the degree of bacterial adherence were determined using a microplate reader.

### Determination of Cell Surface Hydrophobicity

The relative cell surface hydrophobicity of *S. mutans* ATCC 29175 grown in THB-HK ± triclosan at 1/2, 1/4, or 1/8 MIC was determined by measuring their absorption to n-hexadecane according to the procedure described by Rosenberg *et al.* [18].

### RNA Isolation and Quantitative RT-PCR

To investigate the effect of sub-MICs of triclosan on expression of several genes involved in adherence and biofilm formation, *S. mutans* ATCC 25175 was grown to mid-log phase (OD_660_ = 0.45) and then triclosan was added at 1/2, 1/4 or 1/8 MIC prior to further incubate at 37°C for 2 h. Control cells were incubated in the absence of triclosan. Bacteria were collected by centrifugation (7,000×*g* for 5 min) and treated with an RNAprotect bacterial reagent (Qiagen Canada Inc., Montreal, QC, Canada). Bacterial cells were then lysed and RNA was isolated and purified using the RNeasy minikit (Qiagen Canada Inc.). The amounts of mRNA were quantified with the Experion™ system (Bio-Rad Laboratories, Mississauga, ON, Canada). The reverse transcription-polymerase chain reaction (RT-PCR) analysis was performed as follows. RNA from each sample (100 ng/µl) was reverse-transcribed using Maloney murine leukemia virus reverse transcriptase and random hexamers in a Bio-Rad MyCycler™ thermal cycler (Bio-Rad Laboratories). Reverse transcription conditions were 5 min at 70°C, 10 min at 25°C, 50 min at 37°C, and 15 min at 70°C. Real-time PCR was used for quantification of *atlA*, *comD*, *gtfB*, *gtfC*, and *luxS* mRNA expression. 16S rRNA gene was used as an internal control for data normalization. The primers used for the quantitative RT-PCR were purchased from Life Technologies Inc. (Burlington, ON, Canada) and are listed in Table 1. The sequences of primers were obtained from a previous study [19] while the primers for *atlA* were designed in this study. Triplicate reactions were prepared with 25 µl of PCR mixture containing 12.5 µl of IQ SYBR Green Supermix, 5 µl of cDNA, 1 µl of gene-specific primer, and 6.5 µl of RNase- and DNase-free water. The samples were amplified using a Bio-Rad MyCycler™ thermal cycler (Bio-Rad Laboratories). The amplification conditions for *atlA*, *comD*, *gtfC*, *luxS* and 16S rRNA were 95°C for 3 min followed by 30 cycles at 95°C for 45 s, 60°C for 45 s and 72°C for 30 s, while that of *gtfB* was 95°C for 3 min followed by 40 cycles at 95°C for 45 s, 55°C for 45 s and 72°C for 30 s. To validate the specificity of each primer pair, temperature curve analyses were performed.

**Table 1:** Primers used for the quantitative RT-PCR analysis.

Genes	Primer sequences	Product size (bp)
16S rRNA	Sense: 5-CCATGTGTAGCGGTGAAATGC-3′	144
	Antisense: 5′-TCATCGTTTACGGCGTGGAC-3′	
*atlA*	Sense: 5′-TCCAATTGCAGCAAACACAGGA-3′	139
	Antisense: 5′-AGTACTTGCCTGAGACGGAACTGTT-3′	
*comD*	Sense: 5′-TTCCTGCAAACTCGATCATATAGG-3′	113
	Antisense: 5′-TGCCAGTTCTGACTTGTTTAGGC-3′	
*gtfB*	Sense: 5′-AGCCGAAAGTTGGTATCGTCC-3′	123
	Antisense: 5′-TGACGCTGTGTTTCTTGGCTC-3′	
*gtfC*	Sense: 5′-TTCCGTCCCTTATTGATGACATG-3′	122
	Antisense: 5′-AATTGAAGCGGACTGGTTGCT-3′	
*luxS*	Sense: 5′-CCAGGGACATCTTTCCATGAGAT-3′	147
	Antisense: 5′-ACGGGATGATTGACTGTTCCC-3′	

### Statistical Analysis

Unless specified otherwise, assays were run in triplicate and the means ± standard deviations were calculated. Data were analyzed using the Student *t*-test.

## Results

Using a microdilution broth method, the MIC of triclosan for *S. mutans* ATCC 25175 and ATCC 35668 was 7.8 µg/ml. Thereafter, biofilm formation by *S. mutans* was investigated following growth in culture medium ± triclosan at 1/2, 1/4, or 1/8 MIC. As reported in Table 2, biofilm formation by both strains of *S. mutans* was dose-dependently induced by sub-MICs of triclosan, as determined by crystal violet staining. At 1/2 and 1/4 MIC of triclosan, the biofilm of *S. mutans* ATCC 25175 was increased by 6.2- and 5-fold, while that of strain ATCC 35668 was increased by 3- and 2.2-fold, respectively. The biofilm was not significantly affected following growth in the presence of triclosan at 1/8 MIC. The effect of adding 0.25% sucrose to THB-HK on biofilm formation induced by sub-MICs of triclosan was also tested. As shown in Table 2, in the absence of triclosan, an important biofilm was formed by both strains of *S. mutans*. The triclosan sub-MICs-inducing effect on biofilm formation was much less significant in the presence of sucrose. Planktonic cells, estimated by recording the OD_660_ of the bacterial suspensions surrounding the biofilm, were significantly decreased following growth in the presence of 1/2 MIC of triclosan in the presence or not of sucrose (Table 2). Given that the triclosan-induced biofilm formation was optimal for *S. mutans* ATCC 25175 grown in the absence of sucrose, this strain and condition were selected for further analyses.

**Table 2:** Effect of triclosan sub-MICs on biofilm formation and planktonic growth by S. mutans.

Strain	Presence of sucrose	Biofilm formation (A_550_)				Planktonic growth (OD_660_)			
		Control	1/2 MIC	1/4 MIC	1/8 MIC	Control	1/2 MIC	1/4 MIC	1/8 MIC
**ATCC 25175**	−	0.27±0.04	1.68±0.04*	1.33±0.04*	0.36±0.11	0.45±0.12	0.28±0.06*	0.31±0.05	0.34±0.08
	+	1.26±0.07	1.73±0.20*	1.41±0.18	1.19±0.13	0.53±0.08	0.34±0.12*	0.44±0.09	0.59±0.14
**ATCC 35668**	−	0.31±0.06	0.94±0.12*	0.67±0.15*	0.31±0.09	0.57±0.09	0.39±0.04*	0.42±0.14	0.64±0.11
	+	0.74±0.05	0.96±0.09*	0.82±0.14	0.85±0.11	0.65±0.12	0.38±0.10*	0.53±0.15	0.64±0.07

Data are expressed as means ± standard deviations. Controls refer to the absence of triclosan.

* Significantly different at *p*<0.01 compared to control.

**Figure 1:**
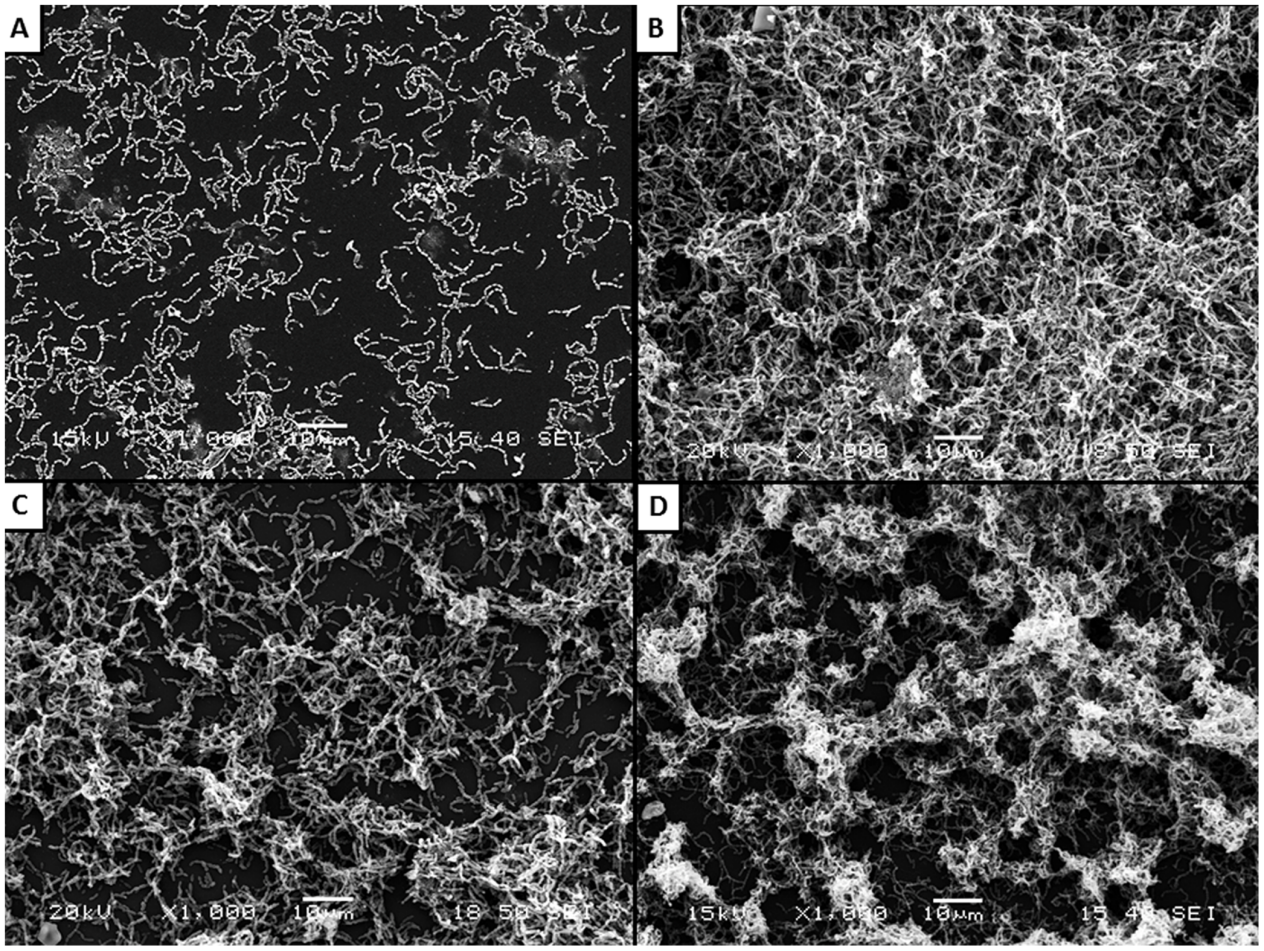
Scanning electron micrographs of *S. mutans* ATCC 25175 biofilm following growth in THB-HK (Panel A) supplemented with 1/2 MIC of triclosan (Panel B), or 1/4 MIC of triclosan (Panel C) or sucrose used as a positive control (Panel D).

Scanning electron microscopy analysis was performed to observe the triclosan sub-MICs-induced biofilm formation by *S. mutans* ATCC 25175. As shown in Figure 1A, individual short chains of *S. mutans* were observed attached to the polystyrene surface when growth was carried out in THB. However, when the culture medium was supplemented with 1/2 and 1/4 MIC of triclosan (Figures 1B and 1C), a thick biofilm made of aggregates and microcolonies of *S. mutans* almost completely covered the surface of the polystyrene support. Sucrose, a well-known biofilm-promoting agent used as positive control, also induced the formation of biofilm (Figure 1D).

**Figure 2:**
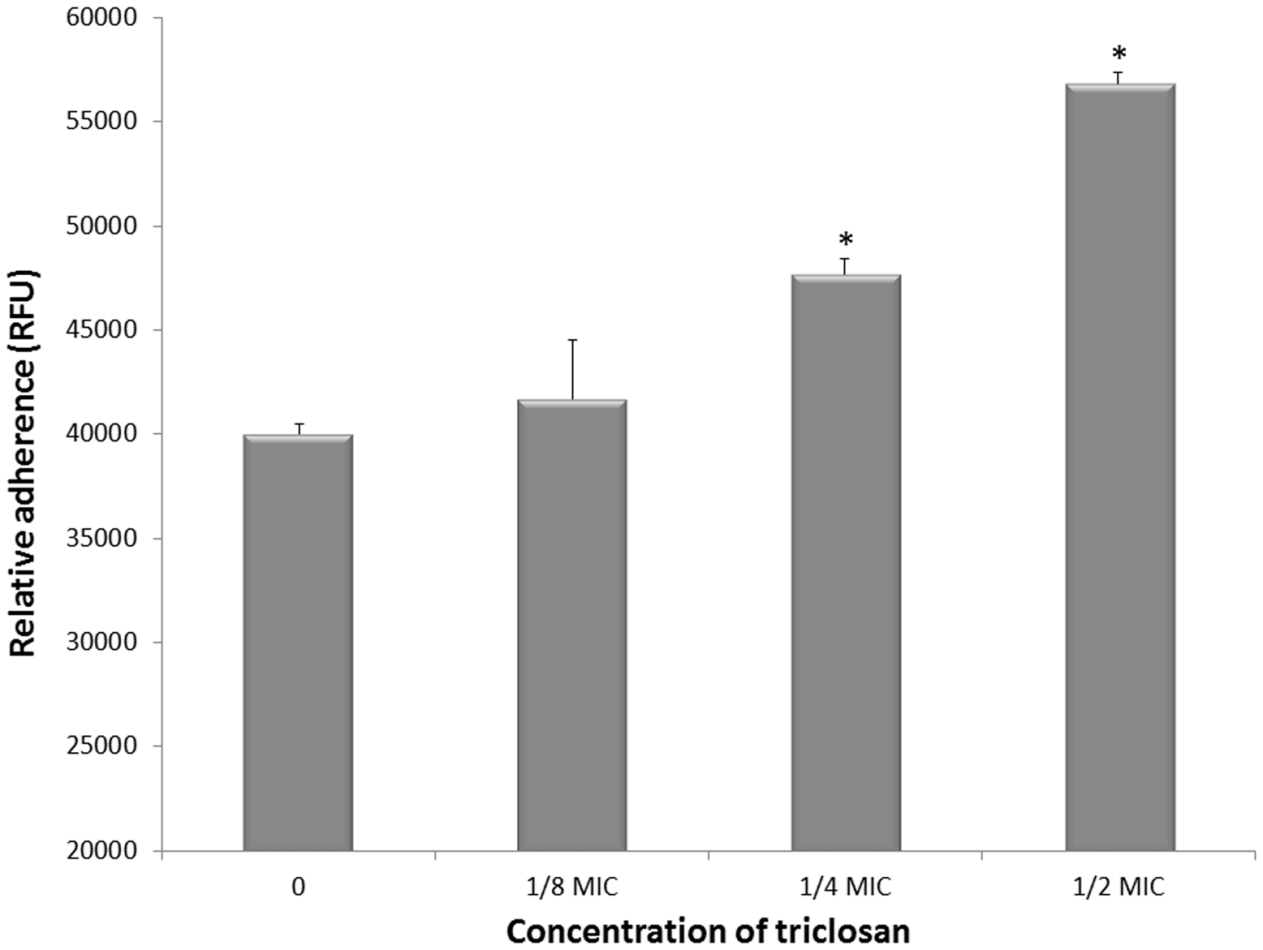
Effect of sub-MICs of triclosan on adherence of *S. mutans* ATCC 25175 to gingival epithelial cells. RFU: Relative Fluorescence Units. Data are expressed as means ± standard deviations. Significant increase (*, *p*<0.01) compared to control bacteria grown in the absence of triclosan.

Thereafter, we further investigated the impact of triclosan at sub-MICs on the host colonization properties of *S. mutans* ATCC 25175 by evaluating the effect on adherence to gingival epithelial cells. As reported in Figure 2, triclosan at 1/2 and 1/4 MIC promoted the adherence of FITC-labeled *S. mutans* to a monolayer of gingival epithelial cells. More specifically, at 1/2 MIC of triclosan, the adherence of *S. mutans* to epithelial cells was increased by 42.5%.

We then attempted to identify the mechanism by which triclosan at sub-MICs may increase the capacity of *S. mutans* to form biofilm and adhere to epithelial cells. Since the hydrophobic properties of the bacterial cell surface may be involved in adherence and biofilm formation, we tested the effect of growing *S. mutans* in the presence of triclosan at sub-MICs on cell surface hydrophobicity. No significant modifications in cell surface hydrophobicity were observed (data not shown).

**Figure 3:**
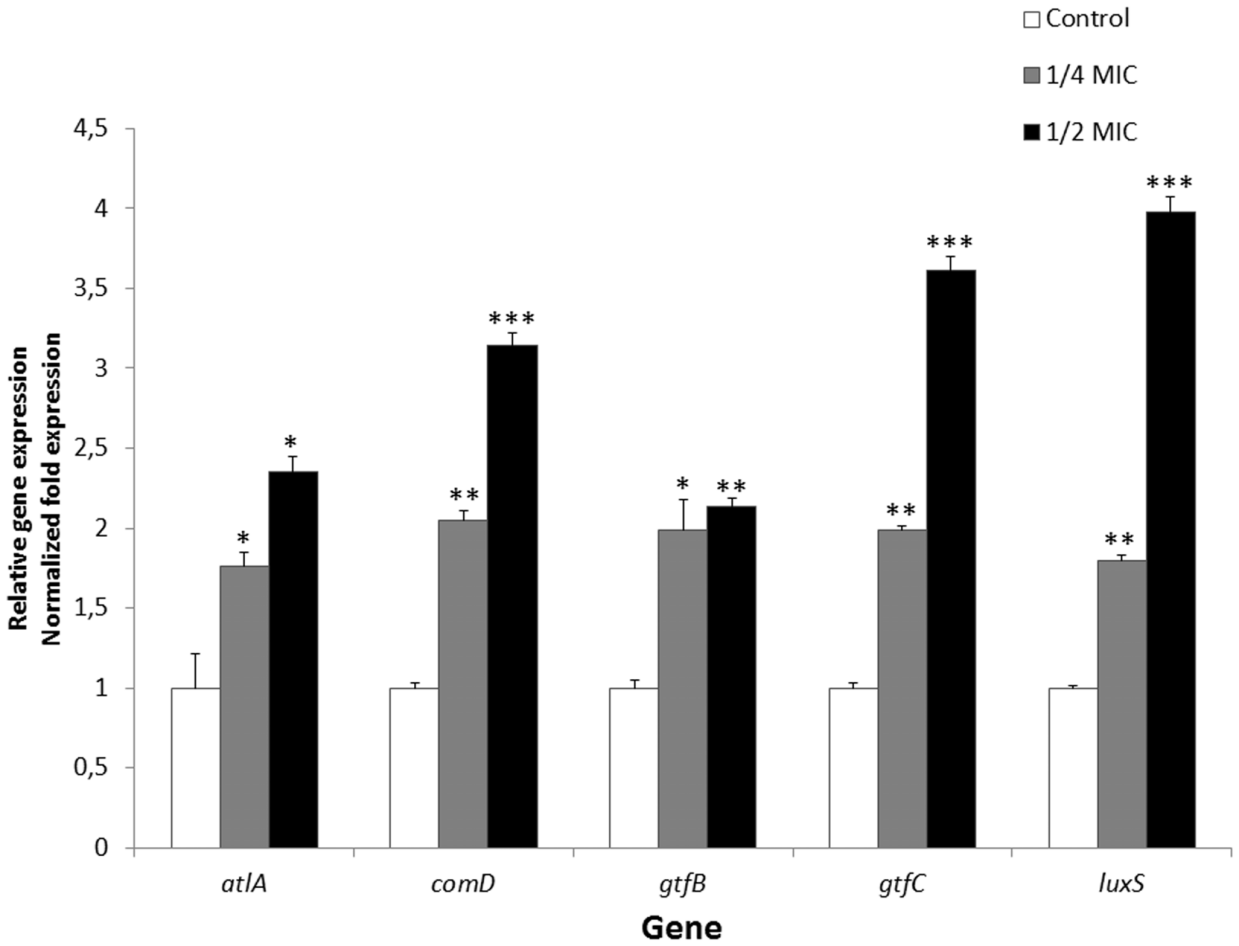
Effect of sub-MICs of triclosan on mRNA expression of specific genes involved in adherence and biofilm formation in *S. mutans* ATCC 25175. Data are expressed as means ± standard deviations. The expression was normalized to 16S rRNA. Significant increase (*, *p*<0.05; **, *p*<0.01; ***, *p*<0.001) compared to untreated control bacteria.

The expression profile of five genes related to adherence and biofilm formation in *S. mutans* was determined following incubation (2 h) of *S. mutans* in the absence and presence of triclosan at 1/2 and 1/4 MIC. As reported in Figure 3, the genes *gtfC* (glucosyltransferase C), *comD* (histidine kinase sensor protein), and *luxS* (autoinducer 2 synthase) were those for which the expression was the most upregulated. More specifically, triclosan at 1/2 MIC, increased *gtfC*, *comD*, and *luxS* expression by 3.6-, 3.1-, and 4-fold, respectively. Although the upregulation of *atlA* (autolysin) and *gtfB* (glucosyltransferase B) expression was less pronounced, it was significantly increased following incubation of *S. mutans* with triclosan at sub-MICs.

## Discussion

Triclosan is a broad spectrum antimicrobial agent used in oral care products to control dental plaque [20]. Although numerous studies investigated the antibacterial properties of triclosan towards oral bacteria [20], [21], there are no data in the literature on the effects of this compound at sub-MICs. Since there are a number of *in vivo* circumstances where concentrations of triclosan may be at subinhibitory levels, we investigated the effects of sub-MICs of this antimicrobial agent on the cariogenic bacterium *S. mutans* in regard to its capacity to colonize the host.

Previous studies have shown that antimicrobial agents at subMICs can either increase or decrease biofilm formation by bacterial pathogens [10], [11], [13], [14]. Our study brought clear evidence that triclosan at sub-MICs significantly increases the biofilm formation capability of *S. mutans*. To the best of our knowledge, this is the first report on the effect of sub-MICs of triclosan on bacterial adherence properties. Prior to our study, only one research group reported on the effect of an antimicrobial agent on biofilm formation by *S. mutans*. More specifically, Dong *et al.* [19] recently showed that sub-MICs of chlorhexidine appear to solidify and strengthen *S. mutans* biofilm. The ability of nicotine to enhance *S. mutans* biofilm formation has also been reported [22].

Although the primary natural location of *S. mutans* is the dental biofilm, we showed that growing *S. mutans* in the presence of triclosan at sub-MICs increased its capacity of adherence to epithelial cells. If the epithelial barriers are breached, adhered *S. mutans* may invade tissue, enter the bloodstream, and ultimately induce infective endocarditis. Since *S. mutans* is an important causative agent of subacute infective endocarditis in particular in subjects with predisposing cardiac conditions [23], further studies should investigate the effects of sub-MICs of triclosan on adherence to endothelial cells.

We then attempted to identify the mechanism by which triclosan at sub-MICs may increase *S. mutans* biofilm formation and adherence to epithelial cells. The cell surface hydrophobicity of bacteria is known to contribute to their adherence properties [24]. Wu *et al.* [25] reported that sub-MICs of specific antibiotics can increase the surface hydrophobicity of another important cariogenic bacterium, *Streptococcus sobrinus*, a phenomenon that may increase their adherence property. In the present study, triclosan at sub-MICs had no effect on the surface hydrophobicity of *S. mutans*, a result that ruled out the involvement of this mechanism in the increased adherence properties of *S. mutans*.

*S. mutans* can use sucrose to synthesize extracellular polysaccharides via glucosyltransferases, more specifically GtfB and GtfC [26]. In this study, although the expression of *gtfB* and *gtfC* was increased in *S. mutans* exposed to triclosan at sub-MICs, it is likely not responsible for the increased biofilm formation observed since sucrose was not used in the culture medium.

Biofilm formation is largely influenced by bacterial communication via quorum-sensing signaling system [27]. More specifically, in *S. mutans*, the *comD* gene product, an histidine kinase sensor protein for the competence-stimulating peptide (CSP), is known to play a critical role in biofilm formation [27]. Moreover, LuxS is produced by many Gram positive bacteria, including *S. mutans*, and is involved in the production of autoinducer 2, another signaling molecule playing a role in biofilm formation [28]. Our study showed that both *comC* and *luxS* genes were significantly upregulated when *S. mutans* was cultivated in the presence of sub-MICs of triclosan. This is likely contributing to the increased biofilm formed under this condition. Dong et al. [19] also reported on the capacity of sub-MICs of antimicrobial agents, more specifically sodium fluoride and tea polyphenols, to increase the mRNA expression of *comD* and *luxS*.

The autolysin AtlA (also known as Smu0630) of *S. mutans* has been reported to play a critical role in biofilm formation regardless of the carbohydrate source. [29]. Interestingly, AtlA has been identified as a fibronectin-binding protein that contributes to bacterial survival in the bloodstream and consequently as a virulence factor for infective endocarditis [30]. This cell surface adhesion whose expression was found to be upregulated in *S. mutans* exposed to sub-MICs of triclosan may contribute to the increased adherence to epithelial cells.

## Conclusions

Our study showed that sub-MICs of triclosan can enhance biofilm formation and epithelial cell adherence of *S. mutans*. We also brought evidence that this may be modulated by an increased expression of specific genes coding for cell surface adhesins or involved in quorum-sensing. Collectively, our data stress the importance of maintaining MIC of therapeutic triclosan to efficiently prevent colonization of the oral cavity by *S. mutans*.
